# Double-barrel STA-MCA bypass and partial trapping of a ruptured mycotic MCA aneurysm with flash fluorescence technique

**DOI:** 10.3171/2021.10.FOCVID21163

**Published:** 2022-01-01

**Authors:** Christopher S. Graffeo, Visish M. Srinivasan, Tyler S. Cole, Michael T. Lawton

**Affiliations:** Department of Neurosurgery, Barrow Neurological Institute, St. Joseph’s Hospital and Medical Center, Phoenix, Arizona

**Keywords:** cerebrovascular bypass, flash fluorescence, mycotic aneurysm, neurosurgical clipping, subarachnoid hemorrhage

## Abstract

Mycotic brain aneurysms are rare and unusual cerebrovascular lesions arising from septic emboli that degrade the elastic lamina and vessel wall of intracranial arteries, which results in pathologic dilatation. Mycotic aneurysms are nonsaccular lesions that are not often suitable for clipping and instead require bypass, trapping, and flow reversal. This case demonstrates the use of indocyanine green “flash fluorescence” to identify the cortical distribution supplied by an aneurysm’s outflow, facilitating safe treatment with a double-barrel extracranial-intracranial bypass and partial trapping and conversion of a deep bypass to a superficial one.

The video can be found here: https://stream.cadmore.media/r10.3171/2021.10.FOCVID21163

## Transcript

This video will illustrate the double-barrel STA-MCA bypass and trapping of a ruptured mycotic MCA aneurysm with the flash fluorescence technique.^1,2^

### 0:33 Patient History and Presentation.

The patient was in his early 50s. His medical history was significant for infectious endocarditis. He recently had a medical status change and was transferred for evaluation. On exam, he was neurologically intact. His diagnostic imaging showed an area of enhancement and T1 hyperintensity concerning for an aneurysm. Angiography confirmed the diagnosis.

### 0:58 Diagnostic Imaging Studies.

Here are those studies. You can see the MRI shows the lesion in the distal insular space, which is the back or posterior portion of the sylvian fissure. The second MRI slice shows an area of hemorrhage in the left frontal lobe. Subsequent angiography showed the mycotic aneurysm with its location at the end of the M2 segment. These are 3D rotational angiograms, showing the complex dolichoectatic morphology of this aneurysm, with one inflow artery and two outflow arteries on the back end.

### 1:34 Positioning, Incision, and First Donor Vessel Harvest.

The patient was taken to the operating room and placed in the supine position with the head oriented horizontally. The craniotomy is shown in blue and extends from the beginning to the end of the sylvian fissure. The skin incision used to expose this flap was a T-shaped incision that was made over the posterior limb of the superficial temporal artery, and “teed” both posteriorly and anteriorly as two separate flaps. The course of the superficial temporal arteries is shown on the illustration to the right, and both of these limbs of the artery were harvested for possible double-barrel bypass.

### 2:07 Surgical Strategy.

Surgical strategy consisted of the following. A T-shaped skin incision that encompassed the full extent of the craniotomy. Harvest of both anterior and posterior branches of the superficial temporal artery as donors. The fronto-temporal-parietal craniotomy, that brought the entire sylvian fissure and the angular recipient artery into view. Anterior and posterior split of the sylvian fissure. Dissection to the aneurysm. Flash fluorescence technique, which involves proximal clipping of the aneurysm inflow, indocyanine green video angiography to identify the outflow territory, and clip removal, with flash fluorescence to illuminate and confirm the outflow territory. Finally, double-barrel STA-MCA anastomoses to the outflow territories. Partial trapping of the middle cerebral artery mycotic aneurysm.^3^

### 3:02 Operative Video Begins and Initial Intradural Exposure.

Here is the incision over the posterior limb of the superficial temporal artery. You can see that the skin incision starts as a T and is extended in both directions, anteriorly and posteriorly. After craniotomy and dural opening, you can see the sylvian fissure, with the frontal lobe to your right and the temporal lobe to your left.

### 3:23 Sylvian Fissure Split and Arterial Inflow Identification.

An aggressive and wide sylvian fissure opening is performed next, following these middle cerebral vessels down into the operculum. You can see the arachnoid adhesions that hold the operculum together, and these are individually cut, and the operculum is widened. Here we continue to follow the vessels down to the insular cortex. And here we see the inflow artery to the aneurysm.

### 3:48 Flash Fluorescence Technique.

Here is our flash fluorescence. You can see that dark territory posteriorly was occluded by the temporary aneurysm clip, but with the release of the clip, you can see the contrast flashing into that closed-off vascular territory.^4–6^

### 4:05 Flash Fluorescence Discussion.

Alright, so the dye comes in. You’ve got a zone of darkness right here. And that was the release of the clip, and you can see the dye sort of “flashing” into that zone. So that’s the whole concept of the “flash fluorescence.” You have to look for a dark spot, and then you release the clip, and you see the dye rush in.

### 4:23 Identification of Recipient Artery and Donor Vessel Triage.

This identifies our recipient angular artery more distally. Here the artery is seen coming out of the sylvian fissure, and a robust recipient is identified. Our superficial temporal artery was not long enough to reach this, and so we had to lengthen the donor vessel by pursuing it into the infrazygomatic space. Here there were some extra loops of the artery that were straightened, and this enabled the artery to then reach all the way back to the angular recipient artery.

### 4:53 First STA-MCA Anastomosis.

The donor vessel was fishmouthed. The donor was loaded with the 10-0 suture, and now our temporary clips are applied. An arteriotomy is made with a right-angle microscissors. And you can see our anchoring sutures here, going first into the heel stitch and second into the toe stitch. Now that the vessels are approximated, we can begin our run of a continuous suture along the first suture line. As we finish, we can tighten the loops of suture and tie our knots. Now the artery is flipped in the opposite direction, and a second continuous running suture line is placed. Here the anastomosis is completed. We come off with our temporary clips, and flow is initiated in the bypass. We can now return to the inflow to the mycotic aneurysm, which we see here, and that inflow artery is now permanently occluded with this aneurysm clip.

### 6:00 First ICG Run.

As a checkup, we can now do an indocyanine green, which confirms patency and flow through the bypass and to the angular artery.

### 6:09 Second STA-MCA Anastomosis.

Here is the recipient vessel also coming out of the aneurysm, and we prepare for our second bypass. Just as the prior bypass, the arteriotomy is made after first temporarily occluding the recipient, our 10-0 sutures are placed to approximate the superficial temporal artery to the recipient, and running continuous sutures are placed along each of the two suture lines. You can see the spacing of our bites, and you can see a nice suture line here on the first side. The artery flips over, and we complete our second line, shown here. Suture is tightened and tied, and our clips come off to initiate flow in the bypass.

### 6:56 Overview of the Completed Bypass Construct.

Here is an overview of our double-barrel STA-MCA bypass, both of which were performed using cortical recipient arteries, thanks to the flash fluorescence technique. Had we not done that, we would have been doing our bypasses deep in the sylvian fissure at the depths of these clips. This is the second partial trapping clip going on the outflow artery from the aneurysm. The aneurysm is now partially trapped, and you can see how in this dominant hemisphere, the Broca and Wernicke areas are nicely protected and preserved.

### 7:28 Detailed Review of Flash Fluorescence Technique.

This demonstrates the flash fluorescence technique. A temporary clip is applied to the artery that fills the aneurysm. With the clip in place, the indocyanine green video angiogram is commenced. Just after the dye reaches the aneurysm, the clip is removed. That dark territory that is originally seen on the cortical surface, shown in gray, then receives a “flash” of dye as the perfusion, then proceeds through the aneurysm, and that flash of contrast confirms that the recipient site on the cortical surface is the target for the bypass.

### 8:05 Clinical Outcome.

The patient awoke with intermittent expressive aphasia. EEG confirmed intermittent temporal lobe seizures, which were controlled with anticonvulsant medications. His aphasia resolved after that, and he returned to his neurological baseline. His infectious endocarditis was managed medically.

### 8:25 Postoperative Imaging Review.

Here are his postoperative imaging studies. The imaging to the left shows, in coronal view, the depth of these clips as they are placed in the insular space. The angiograms to the right show that the aneurysm no longer fills. This next angiogram is an external carotid injection showing our bypasses. The bypass to the left shows the angular artery being filled from the larger of the two donor vessels. The smaller of the two donor vessels can be seen filling that temporal artery. The image to the right is just a later phase view showing good perfusion of these recipient territories. These angiograms show that the aneurysm still fills. The bypass is supplying that outflow artery in a retrograde fashion. It fills all the way down to the aneurysm. There is a little bit of filling of the aneurysm, with recirculation through the aneurysm and perfusion to the outflow vessel. This is an aneurysm that is in the process of thrombosis, and the aneurysm will be protected by this retrograde flow and thrombosis.

### 9:33 Study Conclusions.

In conclusion, lesions posterior to the foramen of Monro require a posterior split of the sylvian fissure. Proximal dissection of the infrazygomatic STA donor extends the usable length of this artery. Mycotic aneurysms are nonsaccular lesions, not often suitable for clipping, and instead require bypass, trapping, and flow reversal.^7^ The “flash fluorescence” technique is an application of IC green technology to identify the cortical distribution supplied by an aneurysm’s outflow and convert the bypass from a deep one to a superficial one. Thank you.

## Acknowledgments

We thank the staff of Neuroscience Publications at Barrow Neurological Institute for assistance with manuscript and video preparation.

## Disclosures

The authors report no conflict of interest concerning the materials or methods used in this study or the findings specified in this publication.

## Author Contributions

Primary surgeon: Lawton. Assistant surgeon: Graffeo, Srinivasan, Cole. Editing and drafting the video and abstract: Lawton, Graffeo, Srinivasan. Critically revising the work: Lawton, Graffeo, Srinivasan. Reviewed submitted version of the work: all authors. Approved the final version of the work on behalf of all authors: Lawton. Supervision: Lawton.
